# Node and edge control strategy identification via trap spaces in Boolean networks

**DOI:** 10.1186/s12859-025-06135-y

**Published:** 2025-10-07

**Authors:** Laura Cifuentes-Fontanals, Elisa Tonello, Heike Siebert

**Affiliations:** 1 Max Planck Institute for Molecular Genetics, Berlin, Germany; 2 Freie Universität Berlin, Berlin, Germany; 3 Kiel University, Kiel, Germany

**Keywords:** Control, Boolean network, Trap space, Node control, Edge control

## Abstract

**Background:**

The study of control mechanisms of biological systems allows for interesting applications in bioengineering and medicine, for instance in cell reprogramming or drug target identification. A control strategy often consists of a set of interventions that, by fixing the values of some components, ensure that the long term dynamics of the controlled system is in a desired state. A common approach to control in the Boolean framework consists in checking how the fixed values propagate through the network, to establish whether the effect of percolating the interventions is sufficient to induce the target state. Although methods based uniquely on value percolation allow for efficient computation, they can miss many control strategies. Exhaustive methods for control strategy identification, on the other hand, often entail high computational costs. In order to increase the number of control strategies identified while still benefiting from an efficient implementation, we introduce the use of trap spaces, subspaces of the state space that are closed with respect to the dynamics, and that can usually be easily computed in biological networks.

**Results:**

This work presents a method based on value percolation that uses trap spaces to uncover new control strategies. It allows for node interventions, which fix the value of certain components, and edge interventions, which fix the effect that one component has on another. The method is implemented using Answer Set Programming, extending an existing efficient implementation of value percolation to allow for the use of trap spaces and edge control. The applicability of the approach is studied for different control targets in a biological case study, identifying in all cases new control strategies.

**Conclusion:**

The method presented here provides a new tool for control strategy identification in Boolean networks that allows for more diversity of interventions and for the possibility of efficiently finding new control strategies that would escape usual percolation-based methods, widening the possibility for potential applications

## Background

Reprogramming a cell to induce a desired cell fate or the identification of drug targets for disease treatment are examples of the multiple applications of the study of control mechanisms in biological systems. Mathematical modeling can help to predict potential control candidates in silico, which might reduce the need for the usually costly and time-consuming experimental testing [1]. Among the different mathematical frameworks, Boolean modeling stands out for its ability to capture the qualitative behavior and dynamics of biological systems even if there is a lack of detailed quantitative data. In the Boolean framework, each component is represented by a binary node that only admits two activity levels, 0 and 1. These two levels might denote, for instance in a gene regulatory network, if a gene is active or not or if the concentration of a certain compound is above or below a certain threshold. The interactions between the components are described by logical functions. Despite its simplicity, Boolean modeling has been shown to reliably capture the relevant dynamics of the modeled biological systems [2, 3].

Control in Boolean networks is a broad field and many different approaches have been developed dealing with different scenarios and goals. Commonly, one wants to influence the system in such a way that the asymptotic dynamics fulfills the desired properties. Thus the focus is on introducing perturbations that influence the system attractors and their reachability properties. Some approaches aim at leading the system towards a desired attractor, from a certain initial state [4] (source-target control) or from any possible initial state [5] (full network control). We refer to this type of control as attractor control. In other cases, only the location of the attractors in a particular subspace is of importance, for instance if a certain phenotype defined by the values of a small set of marker components is the desired outcome. Several approaches have been developed to deal with this control problem, known as target control [6–9].

Since control aims at manipulating dynamical properties of a model, it usually depends on the way the dynamics is derived from the Boolean function representing the system. The so-called synchronous update that updates every component in the model in each step gives rise to deterministic dynamics and is highly amenable to computational analysis. Here, the only cycles in the dynamics are attractors and control can be interpreted as forcing all trajectories starting in an initial state to reach the desired target set. However, non-deterministic asynchronous updates that allow for different (if unknown) time delays of the possible component value changes have been shown to more realistically capture the behavior of biological systems. In this setting, there can be non-attractive cycles in the dynamics that trajectories can leave after entering. In application, trajectories that stay indefinitely in a non-attractive cycle are usually taken as modeling artifacts and are not further considered. Similarly, control approaches neglect such trajectories and solely aim at enforcing attractor properties. Several control methods have been developed specifically for synchronous [10, 11] or asynchronous dynamics [5, 12] while others are applicable to any dynamics [6, 8, 13].

Control interventions can be permanent or temporary, depending on whether the system perturbations are maintained indefinitely or are released after a certain amount of time [14]. One-step control encompasses strategies that apply all the interventions at once, whereas sequential control includes strategies applying interventions at different time steps [4]. Different types of intervention targets can also be considered. Most approaches use interventions that fix the state of a component to a certain value [4, 5, 8, 13]. This type of intervention, called node intervention or node control, can represent for instance the knockout or sustained activation of a gene in a gene-regulatory network. However, sometimes a certain node intervention might not be possible, either because it is unfeasible in practice or because the target component plays a potentially crucial role in some processes that should not be disrupted. In such cases, it is useful to consider interventions targeting only a specific interaction between two components, leaving the rest of the interactions unaltered. This type of intervention is known as edge intervention or edge control. Several approaches exist for identifying edge control strategies, dealing with target control in asynchronous [9] or synchronous dynamics [10, 11].

Multiple approaches have been developed to identify and compute control strategies for Boolean networks in the different settings, using tools ranging from analysis of the stable motifs of the systems [5] to exploiting computational algebra methods [10]. A core idea common to many of them is to utilize value percolation to test the effect of permanently fixing certain component values on the dynamical behavior. Methods based on value percolation can be implemented efficiently [15]. On the other hand, they are quite restrictive and might miss many possible control strategies. To bridge this gap, recent works have dealt with attractor control using basins of attraction [14] or aimed at an exhaustive enumeration of all possible control strategies using model checking queries [13]. However, these approaches might entail high computational resources.

In order to benefit from the efficiency of value percolation and increase the number of identified control strategies, we explore the use of trap spaces for target control. Trap spaces are subspaces of the state space that are closed with respect to the dynamics. Consequently, a trap space contains at least one attractor. Trap spaces are in many cases good approximations of attractors [16] and can be efficiently computed for relatively large networks [17]. Trap spaces can be used as an intermediate control step since by leading the system to a trap space, one ensures that only attractors inside the trap space are reachable. Applying the usual percolation techniques to target trap spaces containing only desirable attractors can potentially uncover new control strategies for both node and edge control.

This article is an extended version of [8], in which we developed a method for computing node control strategies utilizing value percolation in combination with trap space analysis for target control, potentially yielding richer solution sets while keeping computational efficiency. We significantly broaden the theoretical and computational framework to include edge control, reworking the original material to obtain a consistent, comprehensive and flexible approach. For efficient implementation, we use a logical programming approach, namely Answer Set Programming (ASP), extending the works from [15] and [18]. Finally, building on a case study from [8], we show the applicability of the method and illustrate the potential inherent in comprehensive analysis using both node and edge control. The findings presented in this work are included and discussed in context with other approaches to control in LCF’s PhD thesis [19].

We start with a general overview about Boolean modeling. Then, we introduce the different types of interventions considered in this work, node and edge, their effect on the controlled system and the theoretical basis for control strategy identification using value percolation and trap spaces (“Control strategies” section). The implementation of the method using Answer Set Programming is detailed in the “Implementation” section. Finally, we show the applicability of our approach to a cell fate decision network (“Case study: MAPK network”) and compare our results for multiple biological networks to those obtained with alternative control methods (“Comparison”).

### Boolean modeling

We define a *Boolean network* on *n* variables as a function $f = (f_1, \dots , f_n) :\mathbb {B}^n \rightarrow \mathbb {B}^n$, with $\mathbb {B}= \{0,1\}$. The set of variables or components $\{0, \dots n\}$ is denoted by *V*. We call $\mathbb {B}^n$ the *state space* of the Boolean function and every $x \in \mathbb {B}^n$ is a *state* of the state space. Given $i \in V$, $c \in \mathbb {B}$ and $x \in \mathbb {B}^n$, we define ${\bar{x}}_i = 1 - x_i$, ${\bar{c}} = 1 - c$ and ${\bar{x}}^i$ as ${\bar{x}}^i_k = 1 - x_k$ for $k = i$ and ${\bar{x}}^i_k = x_k$ for all $i \ne k \in V$. The *interaction graph* of a Boolean network *f* is defined as the labelled multi-digraph (*V*, *E*) with $E \subseteq V \times V \times \{+,-\}$, admitting an edge from *i* to *j* if there exists $x \in \mathbb {B}^n$, such that $s = (f_j({\bar{x}}^i) - f_j(x))({\bar{x}}^i_i - x_i) \ne 0$. The label of the edge is given by the sign of *s*. Thus, the interaction graph captures the activation (positive) and inhibition (negative) relations between the components of a Boolean network.

The dynamics of a Boolean network is defined by the *state transition graph*, a directed graph with node set $\mathbb {B}^n$. Given a Boolean function *f*, we can define different dynamics depending on the way the components are updated, giving rise to different state transition graphs. For example, in the *synchronous dynamics SD(f)* all the components that can be updated are updated at the same time, whereas in the *asynchronous dynamics AD(f)* only one component is updated at a time. Thus, the synchronous state transition graph has an edge from a state *x* to a state *y* if $x \ne y$ and $f(x) = y$, whereas the asynchronous state transition graph has an edge from a state *x* to a state *y* if $f_i(x) = y_i \ne x_i$ for some $i \in V$ and $x_j = y_j$ for all $i \ne j \in V$. The *generalized asynchronous dynamics GD(f)* includes transitions that update a set of components at a time. Consequently, the corresponding state transition graph has an edge from a state *x* to a state *y* if there exists $\emptyset \ne I \subseteq V$ such that $f_i(x) = y_i \ne x_i$ for $i \in I$ and $x_j = y_j$ for all $i \notin I$. In order to capture the different time scales that might coexist in a biological system, the asynchronous dynamics is often used. The work presented here is valid for any of the three dynamics introduced. We use *D*(*f*) to refer to any of these dynamics.

**Fig. 1:**
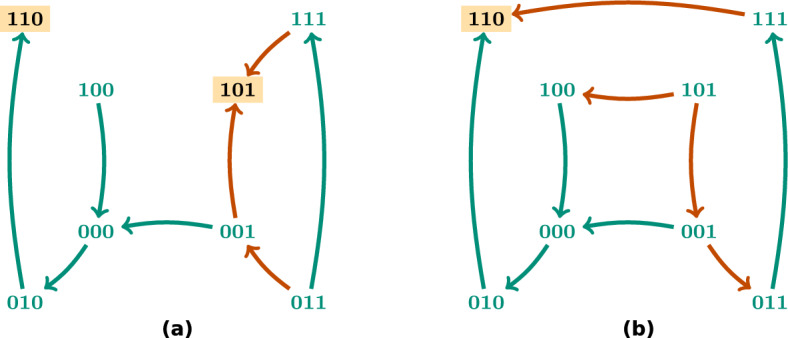
(**a**) Asynchronous dynamics of the Boolean function $f(x) = (x_2 \vee x_3$, $x_2 {\bar{x}}_3 \vee {\bar{x}}_1 {\bar{x}}_3$, $x_2 x_3 \vee x_1 x_3)$, with two attractors $\mathcal {A}_1 = \{110\}$ and $\mathcal {A}_2 = \{101\}$ and six trap spaces (${*}{*}{*}$, ${*}{*}0$, ${*}10$, $1{*}1$, 110 and 101). (**b**) Asynchronous dynamics of the Boolean function $f^{\mathcal {N}}(x) = (x_2$, ${\bar{x}}_1 \vee x_2$, 0) with $\mathcal {N}= \{(3,0)\}$. Transitions that vary between *AD*(*f*) and $AD(f^{\mathcal {N}})$ are marked in red. Attractors are marked in bold. $F(f^{\mathcal {N}})(\mathbb {B}^n) = {*}{*}0$, $\mathbb {B}^n$ does not percolate to *P* under $f^{\mathcal {N}}$ but percolates to the selected trap space $T = {*}{*}0$. Therefore, $\mathcal {N}$ is a control strategy via trap spaces for $P = 110$ in *AD*(*f*). Example adapted from [8] used with permission from the publisher

The long term dynamics of the system is captured by the attractors. An *attractor* is a minimal trap set, that is, a minimal set of states that is closed with respect to the dynamics. Attractors correspond to the terminal strongly connected components in the state transition graph. An attractor $\mathcal {A}\subseteq \mathbb {B}^n$ is called *steady state* when $|\mathcal {A}| = 1$ and * complex attractor* when $|\mathcal {A}| > 1$. Fig. 1a shows an example of an asynchronous dynamics for a Boolean network with three components with two steady states. Steady states in a biological system might be associated with different cell fates or cell types and complex attractors with different cell cycles or cell processes with oscillatory behaviours.

Given a set of components $I \subseteq V$ and a state $d \in \mathbb {B}^n$, the *subspace induced by I and d* is defined as $\Sigma (I,d) = \{ x \in \mathbb {B}^n \ | \ x_i = d_i$ for all $i \in I \}$. We denote subspaces by writing the value 0 or 1 for the fixed variables and ${*}$ for the free ones. For example, the subspace ${*}{*}10$ denotes the set of states $\{ x \in \mathbb {B}^n \ | \ x_3 = 1$ and $x_4 = 0 \}$. We define the size of a subspace as the number of fixed variables. A subspace that is closed with respect to the dynamics is a *trap space*. The asynchronous dynamics in Fig. 1a has six trap spaces: ${*}{*}{*}$, ${*}{*}0$, ${*}10$, $1{*}1$, 110 and 101. Trap spaces are invariant with respect to the type of update, contrary to attractors and trap sets, which might be different in different dynamics.

## Results

### Control strategies

This work deals with target control and considers two types of interventions: node interventions and edge interventions. Node interventions fix a certain component to a certain value. More formally, a node intervention (*i*, *c*), with $i \in V$ and $c \in \mathbb {B}$, sets the component *i* and its regulatory function $f_i$ to the value *c*. This type of intervention can be seen, for example in a gene regulatory network, as the knock-out or permanent activation of a gene.

In the context of practical applications in biological systems, a potential limitation of node interventions might be that they necessarily affect all the regulations depending on the controlled node. For this reason, more focused control interventions, for instance interventions acting only on the interaction between two components, are being considered [20]. This type of interventions, which would only alter the effect of a specific component on another without affecting the rest, are known as edge interventions. More formally, an edge intervention (*i*, *j*, *c*), with $i,j \in V$ and $c \in \mathbb {B}$, fixes the value of the component *i* in the regulatory function $f_j$ to the value *c*. By definition, when a regulatory function $f_j$ depends on a component *i*, there exists an edge from *i* to *j* in the interaction graph. An edge intervention can be seen as the deletion of such an edge in the interaction graph, since $f_j$ does not depend on the component *i* after the intervention. In a biological system, an edge intervention can represent for example the modification of a protein that prevents it from binding to a certain component, while still allowing it to interact with the rest of the system.

Given a Boolean network *f* and a set of interventions $\mathcal {C}$, which might be node or edge interventions, we consider the simultaneous application of all interventions in $\mathcal {C}$ on *f*. We write $f^{\mathcal {C}}$ for the function resulting from the application of the interventions in the set $\mathcal {C}$. In the first section we give the formal definition for the function $f^{\mathcal {C}}$ and a control strategy. In the second section, we recall properties of value percolation and establish the basis for control strategy identification for node and edge control.

#### Controlled networks and control strategies

We start by establishing the basic conditions that a set of interventions needs to satisfy in order to be consistent. These conditions aim at preventing, for instance, that a node intervention fixes a component to 1 while another is fixing the same component to 0.

Consider $\mathcal {N}\subseteq V \times \mathbb {B}$ and $\mathcal {E}\subseteq V \times V \times \mathbb {B}$. We call $\mathcal {C}= \mathcal {N}\cup \mathcal {E}$ a *consistent* set of interventions if the following conditions are satisfied:

(i) for all $i,j \in V$ and $c,c' \in \mathbb {B}$, if $(j,c) \in \mathcal {C}$, then $(i,j,c') \notin \mathcal {C}$;
(ii) for all $i,j \in V$ and $c,c' \in \mathbb {B}$, if $(i,c) \in \mathcal {C}$, then $(i,{\bar{c}}) \notin \mathcal {C}$ and $(i,j,c') \notin \mathcal {C}$;
(iii) for all $i,j \in V$ and $c \in \mathbb {B}$, if $(i,j,c) \in \mathcal {C}$, then $(i,j,{\bar{c}}) \notin \mathcal {C}$.

The first condition ensures that node and edge interventions do not act on the same target. The second guarantees that when a node intervention fixes the value of a component, no other intervention fixes that component. The last one prevents edge interventions from fixing the same component to different values in the same regulatory function.

In order to describe the effect of node and edge interventions in a Boolean network, we first define a function $h^{j,\mathcal {C}} :\mathbb {B}^n \rightarrow \mathbb {B}^n$ that, given a set of consistent interventions $\mathcal {C}$ and a component $j \in V$, captures the effect of fixing the components involved in the interventions acting on the regulatory function of *j*. For every $x \in \mathbb {B}^n$, $i,j \in V$, we set

$$\begin{aligned} h^{j,\mathcal {C}}(x)_i = \left\{ \begin{array}{ll} c & \text {if } (i,c) \in \mathcal {C} \text { or } (i,j,c) \in \mathcal {C} \text { for some } c \in \mathbb {B}, \\ x_i & \text {otherwise}. \end{array} \right. \end{aligned}$$

Note that $h^{j,\mathcal {C}}$ is well-defined when $\mathcal {C}$ is consistent. Given a Boolean network *f* and a consistent set of interventions $\mathcal {C}$, we can now define the controlled network $f^{\mathcal {C}}$. For every $k \in V$,

$$\begin{aligned} f_k^{\mathcal {C}} = \left\{ \begin{array}{ll} c & \text {if } (k,c) \in \mathcal {C} \text { for some } c \in \mathbb {B}, \\ f_k \circ h^{k, \mathcal {C}} & \text {otherwise}, \\ \end{array} \right. \end{aligned}$$

where $\circ$ denotes the usual function composition.

The interventions considered in node control fix certain components (nodes) to certain values. Thus, if a set of interventions consists exclusively of node interventions ($\mathcal {C}= \mathcal {N}$), then it can be associated with a subspace $\Sigma (I,d)$, with $I \subseteq V$ and $d \in \mathbb {B}^n$ such that $(i,d_i) \in \mathcal {N}$ if and only if $i \in I$ [8]. When all interventions are edge interventions ($\mathcal {C}= \mathcal {E}$) the controlled regulatory function for any component *k* is given by $f^{\mathcal {E}}_k = f_k \circ h^{k, \mathcal {E}}$.

Given a node intervention (*i*, *c*), one could consider a set of edge interventions that fix the regulatory function $f_i$ to *c*. For instance, if a node *i* has only one incoming edge from *j* in the interaction graph of *f*, that is, the regulatory function satisfies $f_i(x) = x_j$ or $f_i(x) = {\bar{x}}_j$, the node intervention (*i*, *c*) is equivalent in the long-term dynamics to the edge intervention (*j*, *i*, *c*) or $(j,i,{\bar{c}})$ respectively. Note that a node intervention might have multiple equivalent sets of edge interventions. For example, if $f_i(x) = x_j \vee x_k$ for some $i,j,k \in V$, using either the edge intervention (*j*, *i*, 1) or the edge intervention (*k*, *i*, 1) would have the same long-term effect in the dynamics as the node intervention (*i*, 1).

##### Definition 1

Given a Boolean network *f* and a subspace $P \subseteq \mathbb {B}^n$, a set of interventions $\mathcal {C}$ is a *control strategy for the target P* in *D*(*f*) if $\mathcal {A}\subseteq P$ for any attractor $\mathcal {A}$ of $D(f^{\mathcal {C}})$.

A set of interventions defines a control strategy for a given target when all the attractors of the controlled network are contained in the target. When considering only node control, since a set of node interventions $\mathcal {N}$ defines a subspace, a control strategy can also be identified with the subspace associated with $\mathcal {N}$ (see [8, 13]).

We define the size of a control strategy $\mathcal {C}$ as the number of interventions $|\mathcal {C}|$. In the case of $\mathcal {E}= \emptyset$, the number of interventions corresponds to the number of fixed variables. In practical applications, we are interested in intervention sets that are minimal with respect to inclusion. This is a natural approach when considering interaction sets that contain only node interventions or only edge interventions. For simplicity, in this work we use the same definition of minimality for intervention sets that mix edge and node interventions. Depending on the context, the resources required to implement different interventions can vary, and more sophisticated objective functions might take these differences into account.

**Fig. 2:**
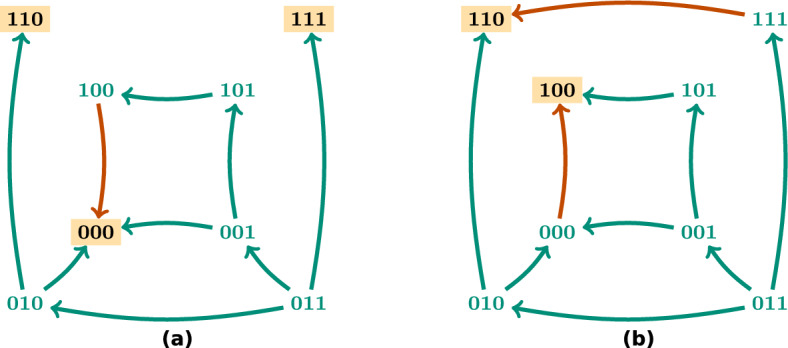
(**a**) Asynchronous dynamics of the Boolean function $$f(x) = (x_2 \vee x_3$$, $$x_1 x_2$$, $$x_1 x_2 x_3)$$. (**b**) Asynchronous dynamics of the Boolean function $$f^{\mathcal {E}}(x) = (1, x_1 x_2, 0)$$, with $$\mathcal {E} = \{(2,1,1), (2,3,0)\}$$. Transitions that vary between *AD*(*f*) and $$AD(f^{\mathcal {E}})$$ are marked in red. Attractors are marked in bold. $$\mathcal {E}$$ is a control strategy for $$P = 1{*}0$$. There is no control strategy for *P* consisting only of a node intervention on the second component

An example of a control strategy using node interventions is shown in Fig. 1, where the set $\mathcal {N}= \{(3,0)\}$, associated with the subspace ${*}{*}0$, is a control strategy for the one-element target subspace $P = 110$, since $f^{\mathcal {N}}$ only has one attractor that is the steady state 110. Figure 2 shows an example where the set of edge interventions $\mathcal {E} = \{(2,1,1),(2,3,0)\}$ is a control strategy for the target $P = 1{*}0$. Note that, if we do not allow interventions on the variables fixed in the target *P*, there are no node control strategies for *P*, since $000 \notin P$ is a steady state of $f^{\mathcal {N}_0}$, with $\mathcal {N}_0 = \{(2,0)\}$, and $111 \notin P$ is a steady state of $f^{\mathcal {N}_1}$, with $\mathcal {N}_1 = \{(2,1)\}$. Thus, in this scenario, control can only be achieved by using edge interventions. This example illustrates how edge interventions can broaden the possibilities for control.

#### Value percolation and control strategy identification

In the following, we recall the concept of value percolation and some properties of percolated subspaces and trap spaces that are helpful in the identification of control strategies.

**Fig. 3:**
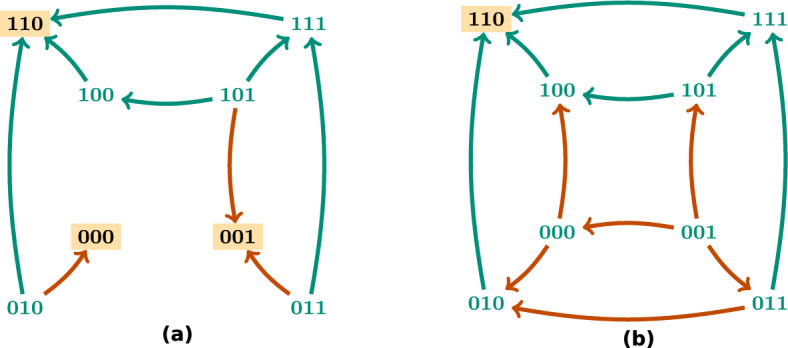
Asynchronous dynamics of the Boolean function $f(x) = (x_2 \vee x_1 {\bar{x}}_3$, $x_1$, ${\bar{x}}_1 x_3)$ (left) and $f^{\mathcal {N}}(x) = (1$, 1, 0) with $\mathcal {N}= \{(1,1)\}$ (right). Transitions that vary between *AD*(*f*) and $AD(f^{\mathcal {N}})$ are marked in red. Attractors are marked in bold. $\mathcal {N}$ is a control strategy for $P = 110$ in *AD*(*f*). $\mathbb {B}^n$ percolates to *P* under $f^{\mathcal {N}}$

Value percolation is based on the idea that fixing some components might cause other components to get fixed downstream. For instance, in the example of Fig. 3, when fixing the first component to 1, the second and third components will get fixed to 1 and 0 respectively since $f_2(x) = 1$ and $f_3(x) = 0$ for all *x* such that $x_1 = 1$, that is, $x \in 1{*}{*}$.

Given a Boolean function *f*, we define the *percolation function* with respect to *f* as the function $F(f) :\mathcal {S} \rightarrow \mathcal {S}$, where $\mathcal {S}$ is the set of all subspaces in $\mathbb {B}^n$, that maps a subspace $S \in \mathcal {S}$ to the smallest subspace that contains the image of *S* under *f*, *f*(*S*), with respect to inclusion. That is, given a subspace $S \in \mathcal {S}$, $F(f)(S) = \Sigma (I,d)$ with $I = \{i\in V\ |\ |f_i(S)| = 1\}$ and $d \in \mathbb {B}^n$ such that $d_i = f_i(x)$ for all $x \in S$ for $i \in I$. Given two subspaces $S,S' \subseteq \mathbb {B}^n$, we say that the subspace *S* *percolates to *$S'$ *under f* if and only if there exists $k \ge 0$ such that $F(f)^k(S) = S'$.

For a set of interventions $\mathcal {C}$, $F(f^{\mathcal {C}})$ captures the propagation of the fixed values through the network. We can use the definition of percolation to formalise the notion of equivalence between intervention sets: we say that two intervention sets $\mathcal {C}_1$ and $\mathcal {C}_2$ are *equivalent* if $F(f^{\mathcal {C}_1})(\mathbb {B}^n) = F(f^{\mathcal {C}_2})(\mathbb {B}^n)$.

Note that if *T* is a trap space, $T' = F(f)(T)$ is also a trap space and $T' \subseteq T$. Moreover, for every $x \in T$ there exists a path in *D*(*f*) from *x* to some $y \in T'$. Detailed proofs of these properties of subspace percolation can be found in [13]. A consequence of these observations is that, given a trap space *T* that percolates to a subspace *S*, there cannot be an attractor $\mathcal {A}\subseteq T$ that is not contained in *S*, since for every state $x \in T$ there exists a path to some $y \in S$. Taking $T = \mathbb {B}^n$, we derive the following result.

##### Proposition 2

Let $P \subseteq \mathbb {B}^n$ be a subspace and *f* a Boolean function. Let $\mathcal {C}$ be a set of interventions such that $\mathbb {B}^n$ percolates to *P* under $f^{\mathcal {C}}$. Then $\mathcal {C}$ defines a control strategy in *D*(*f*) for *P*.

We refer to the control strategies satisfying the conditions of Proposition 2 as *control strategies by direct percolation*. An example of such a control strategy is shown in Fig. 3. This type of control strategy requires, in general, that the interventions are applied permanently. Note that permanent interventions may induce the creation of new attractors. Thus, control strategies by direct percolation are useful when the definition of the control problem does not require the dynamics to evolve to an attractor of the original network but rather to any attractor that is contained in the target subspace. Several approaches to the identification of control strategies by direct percolation using node control have been developed [6, 7] and there exist implementations that identify all control strategies by direct percolation efficiently [15]. However, there are still many control strategies that do not fulfill the conditions of Proposition 2. Figure 1 shows an example of control strategy that does not percolate to the target subspace.

In order to exploit the efficiency of value percolation to identify more control strategies, we developed a method based on percolation that uses trap spaces [8]. As mentioned before, trap spaces are subspaces closed for the dynamics. Thus, each trap space contains at least one attractor. From all trap spaces of a Boolean function, we select the ones that contain only attractors belonging to the target subspace. We call such trap spaces *selected trap spaces*. Proposition 3 introduces sufficient conditions for a subspace to be a control strategy for a target via a selected trap space.

##### Proposition 3

Let $P \subseteq \mathbb {B}^n$ be a subspace and *f* a Boolean function. Let $T = \Sigma (I,d)$ be a trap space such that if $\mathcal {A}\subseteq T$ is an attractor of *D*(*f*), then $\mathcal {A}\subseteq P$. Let $\mathcal {C}$ be a set of interventions such that $\mathbb {B}^n$ percolates to *T* under $f^{\mathcal {C}}$ and for all $(i,c) \in \mathcal {C}$, $i \in I$ and for all $(i,j,c) \in \mathcal {C}$, $j \in I$. Then $\mathcal {C}$ defines a control strategy in *D*(*f*) for *P*.

##### Proof

Let $\mathcal {A}\subseteq \mathbb {B}^n$ be an attractor for $D(f^{\mathcal {C}})$. Since $\mathbb {B}^n$ percolates to *T* under $f^{\mathcal {C}}$, for every $x \in \mathbb {B}^n$, in particular for every $x \in \mathcal {A}$, there exists a path in $D(f^{\mathcal {C}})$ from *x* to some $y \in T$. Therefore, $\mathcal {A}\subseteq T$. Since $\mathbb {B}^n$ percolates to *T* under $f^{\mathcal {C}}$, *T* is also a trap space in $f^{\mathcal {C}}$, so $f^{\mathcal {C}}_k(x) = d_k = f_k(x)$ for all $k \in I$ and $x \in T$. Since for all $(i,c) \in \mathcal {C}$, $i \in I$ and for all $(i,j,c) \in \mathcal {C}$, $j \in I$, we have that $f^{\mathcal {C}}_k(x) = f_k \circ h^{k,\mathcal {C}} (x) = f_k(x)$ for all $k \notin I$ and $x \in T$. Consequently, $f^{\mathcal {C}}(x) = f(x)$ for all $x \in T$. Since $\mathcal {A}\subseteq T$ and for all $x \in T, f(x) = f^{\mathcal {C}}(x)$, $\mathcal {A}$ is also an attractor of *D*(*f*) and, therefore, $\mathcal {A}\subseteq P$. $\square$

In the case $\mathcal {C} = \mathcal {N}$, the condition of $i \in I$ for all $(i,c) \in \mathcal {C}$ corresponds to $T \subseteq \Omega$, where $\Omega$ is the subspace associated to $\mathcal {N}$ [8].

**Fig. 4:**
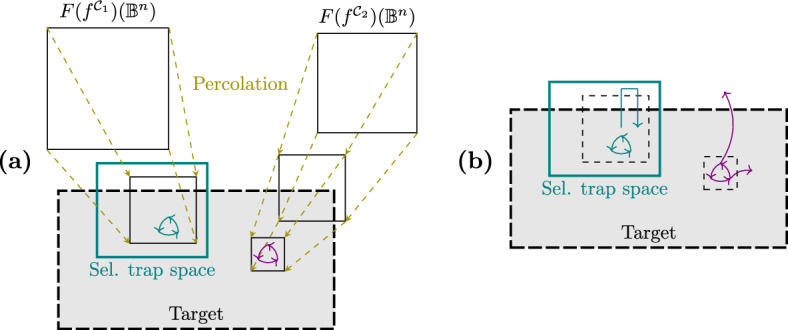
(**a**) Illustration of the ideas behind the methods for control strategy identification by direct percolation and via trap spaces. Rectangles represent subspaces, using solid lines in case of trap spaces and dashed lines otherwise. The yellow dashed lines represent the percolation process, with the state space percolating towards the target under two different control strategies ($\mathcal {C}_1$ and $\mathcal {C}_2$). The three-arrow cycles represent attractors. The selected trap space used for control via trap spaces is marked in green. (**b**) Possible trajectories of the system once the control is released. Note that none of the trajectories starting in a state belonging to the selected trap space can reach any attractor outside the target subspace (green arrows) whereas that might be possible in trajectories starting at any other point within the target subspace (purple arrows)

We call the control strategies satisfying the conditions of Proposition 3*control strategies via trap spaces*. Note that control strategies via trap spaces, contrary to control strategies by direct percolation, cannot introduce new attractors. All the attractors of the controlled network are attractors of the original system, since the dynamics within the trap space is preserved. Fig. 1 shows an example of this type of control strategy. $T = {*}{*}0$ is a selected trap space for the target $P = 110$, since it contains only the attractor $\mathcal {A}= 110$. $\mathbb {B}^n$ percolates to *T* under $f^{\mathcal {N}}$, with $\mathcal {N}= \{(3,0)\}$, since $F(f^{\mathcal {N}})(\mathbb {B}^n) = {*}{*}0$. Consequently, $\mathcal {N}$ is a control strategy via trap spaces for *P*. Since $\mathbb {B}^n$ does not percolate to *P* under $f^{\mathcal {N}}$, $\mathcal {N}$ is not a control strategy by direct percolation. Figure 4 illustrates the main idea of the methods for control strategy identification by direct percolation and via trap spaces.

We can easily identify all the selected trap spaces if the attractors of the Boolean network are known or, alternatively, if they can be approximated by minimal trap spaces [16], that is, if each minimal trap space contains only one attractor and every attractor is included in a minimal trap space. Although attractor identification can be hard to achieve depending on the particular problem, the second property is easier to verify and is relatively common in Boolean networks modeling biological systems [16].

Control strategies by direct percolation do not depend on the update. On the other hand, the selected trap spaces are defined in terms of the attractors, which might vary in different updates. As a consequence, control strategies via trap spaces are also in general update-dependent. This provides the method with enough flexibility to identify control strategies that are valid in one update but not in another.

A further advantage of the control strategies identified by Proposition 3 is that they allow for the control interventions to be eventually released. Once a selected trap space is reached, the system will remain in the trap space, regardless of whether the control interventions are active or not (see Fig. 4). Moreover, if the dynamics has already stabilized, since all the attractors of the controlled network are also attractors in the original network, a release of the control would not alter the state of the system. Thus, control strategies via trap spaces can be applied using permanent interventions as well as temporary ones. This additional property widens the range of possible choices for control since interventions relying on agents that decay over time could also be considered.

Although the methods using direct percolation and percolation via trap spaces are both based on value propagation, the control strategies that they identify can be very different. For instance, control strategies by direct percolation might introduce new attractors on the controlled system, while control strategies via trap spaces preserve the original attractors within the selected trap space. Moreover, the interventions used in control via trap spaces must target one of the components fixed in the selected trap space, while control by direct percolation has no restriction on the interventions. Even when the candidate strategy consists of interventions targeting components fixed in a selected trap space, the controlled system could still percolate directly to the target subspace but not to the selected trap space. As a consequence, in many cases, there might be control strategies that are obtained by direct percolation that are not identified via trap spaces and vice versa. For this reason, it is useful to use the two methods in combination (see “Target: apoptotic phenotype” for an example of this scenario).

### Implementation

The methods for control strategy identification presented in this work are based on the identification of sets of interventions that cause the state space to percolate either to the target subspace or to one of the selected trap spaces under the controlled function. Identifying all the minimal control strategies of this type might entail the exploration of all possible sets of interventions, whose number grows exponentially with the size of the network (for node control) or with the number of edges (for edge control).

The use of Answer Set Programming (ASP) was proposed by Kaminski et al. [15] to deal with the combinatorial explosion associated with node control. Answer Set Programming is a form of declarative programming that works well with hard combinatorial, search and optimization problems. This type of problems often entail a decision-making process over a set of candidates to decide whether they satisfy a specified constraint and possibly identify an optimised output. In order to solve a problem with ASP, one needs to provide a description of the problem using logical rules. Solving the original problem is then reduced to identifying the solutions of its corresponding logic program. For a more extensive explanation of Answer Set Programming and its semantics we refer the reader to [21].

In [18] we extended the work done in [15] to identify the control strategies presented in [8]. In this work, we recall the implementation of [18] and extend it to deal with edge control. A detailed description of the full ASP encoding is available in “Methods”.

#### Outline of the algorithm

The complete algorithm for control strategy identification is shown in Algorithm 1. It combines and extends the algorithms introduced in [8] and [18]. Algorithm 1 takes as inputs the Boolean function *f*, the target subspace *P*, the type of control method *method*, the limit size for the control strategies *k*, the (possibly empty) list of forbidden interventions $avoid\_intvs$ and, optionally, the list of attractors *attr* (line 1). The Boolean function, target subspace, selected trap spaces, limit size and list of forbidden interventions are used as input for the ASP program, which is called by *createCandidatesAndPercolate* (lines 3, 10, 12) and returns the corresponding control strategies. Our ASP encoding takes as input a constant-free Boolean function. Therefore, if the network has constant coordinate functions, a preprocessing step takes place so that constant values are percolated and removed from the network. Note that this does not affect the attractors of the network, as explained in “Control strategies”.

Algorithm 1 allows for the computation of control strategies by direct percolation (lines 2–3), via the trap spaces method (lines 9–10) and using the two methods combined, meaning that both percolation to the target subspace and selected trap spaces is considered (lines 11–12). When searching for control strategies via the trap spaces, we distinguish two types of selected trap spaces: trap spaces contained in *P* (Type 1) (line 6) and trap spaces not contained in *P* but containing only attractors in *P* (Type 2) (line 8). Note that selected trap spaces of Type 2 are only identified when all the attractors are known or can be approximated by minimal trap spaces (line 7). Moreover, in order to avoid unnecessary calculations, we only consider non-percolating trap spaces, that is, trap spaces that do not percolate to smaller ones, since all the subspaces percolating to a trap space *T* also percolate to *F*(*f*)(*T*).

We implemented Algorithm 1 using PyBoolNet [22], a Python package for the generation, modification and analysis of Boolean networks. PyBoolNet also provides an efficient computation of trap spaces for relatively large networks, which we use for the computation of the selected trap spaces, and a method to check whether the attractors of a Boolean network can be approximated by minimal trap spaces [16]. To solve the ASP problem, we use *clingo*, included in Potassco, the Potsdam Answer Set Solving Collection [23].

### Considerations on minimality and running times

Since we are interested in minimal intervention sets, the ASP program is run to return all the minimal sets of interventions with respect to inclusion that are control strategies by direct percolation and/or via trap spaces up to the chosen limit size. The output set of control strategies might vary depending on the method that is chosen and consequently there might be control strategies that are minimal by direct percolation and not minimal via trap spaces and vice versa. For instance, for the Boolean network of Fig. 1 and the target $P = 110$, there is only one minimal control strategy for node control via trap spaces, the set $\mathcal {N}_1 = \{(3,0)\}$. Direct percolation instead identifies the unique minimal control strategy $\mathcal {N}_2 = \{(2,1), (3,0)\}$. When computing the control strategies combining both methods, only $\mathcal {N}_1$ will be identified, since $\mathcal {N}_2$ is a superset of $\mathcal {N}_1$. For this reason, the set of control strategies identified by the combined method is not necessarily equivalent to the union of the control strategies obtained by each method, even though each control strategy is identified either by direct percolation or via trap spaces. This is the case in the biological network analysed in “Case study: MAPK network”, where some of the control strategies identified by direct percolation are non-minimal and therefore not present as control strategies of the combined method since they are supersets of a smaller one.

The main factors influencing the running times of the approach are the size and complexity of the network (number of nodes, edges, prime implicants, etc.) and the number and size of the target subspaces and selected trap spaces.

Assuming that there are no nodes nor edges to avoid, the amount of candidate interventions to choose from for direct percolation is twice the number of nodes 2*n* for node control, twice the number of edges 2*m* for edge control or twice the sum of both $2(n+m)$ for the mixed control. Thus, the total number of possible combinations grows exponentially with 2*n*, 2*m* or $2(n+m)$ respectively. In order to reduce the number of candidates, the previous works [6] and [15] only consider a candidate node intervention (*i*, *c*) when there is a positive (respectively negative) path from *i* to one of the nodes fixed in the target subspace to the value *c* (respectively ${\bar{c}}$). See [6] and [15] for a full explanation of this reduction. The efficiency of the ASP approach for node control was analysed in [15], where the running times of different Boolean networks and different ASP solvers were studied. The number of edges could, in theory, be as large as $n^2$, however this is not usually the case in biological systems, which are often rather sparse. Although extending the candidate interventions to edges has an impact on the running times (see “Running times” section), the problem is still treatable for relatively large biological networks.

In the case of percolation via trap spaces, the number of candidate interventions might be reduced since, according to Proposition 3, the intervention candidates are required to target only variables fixed in the corresponding selected trap space. Consequently, the running times are also dependent on the number and size of the selected trap spaces. Note that when the selected trap space is a steady state, the number of candidate interventions is the same as in the method of direct percolation.

In the example of Fig. 1, taking $P = 110$ as the target subspace, there are 8, 577 and 656 consistent candidate combinations for node, edge and mixed control respectively for the direct percolation method. When considering the method via trap spaces, since one of the selected trap spaces is a steady state ($T_1 = 110$), the amount of candidate interventions is the same. If only $T_2= {*}{*}0$ was considered as a selected trap space, the number of combinations of candidate interventions would be 2, 27 and 28 for node, edge and mixed control respectively, since they would only include node and edge interventions targeting the third component.

The last factor to take into account when analysing the running times is the number of candidate subspaces, both in case of direct percolation (if the target is extended to multiple subspaces) and in the case of percolation via trap spaces when different selected trap spaces act as targets. The more target subspaces, the more likely that a candidate intervention satisfies the condition. However, increasing the number of target subspaces might also increase the number of candidate interventions. Moreover, in some cases, the combined method might require higher running times than the sum of the times required for direct percolation and via trap spaces individually (see results in the following section).

### Case study: MAPK network

**Fig. 5:**
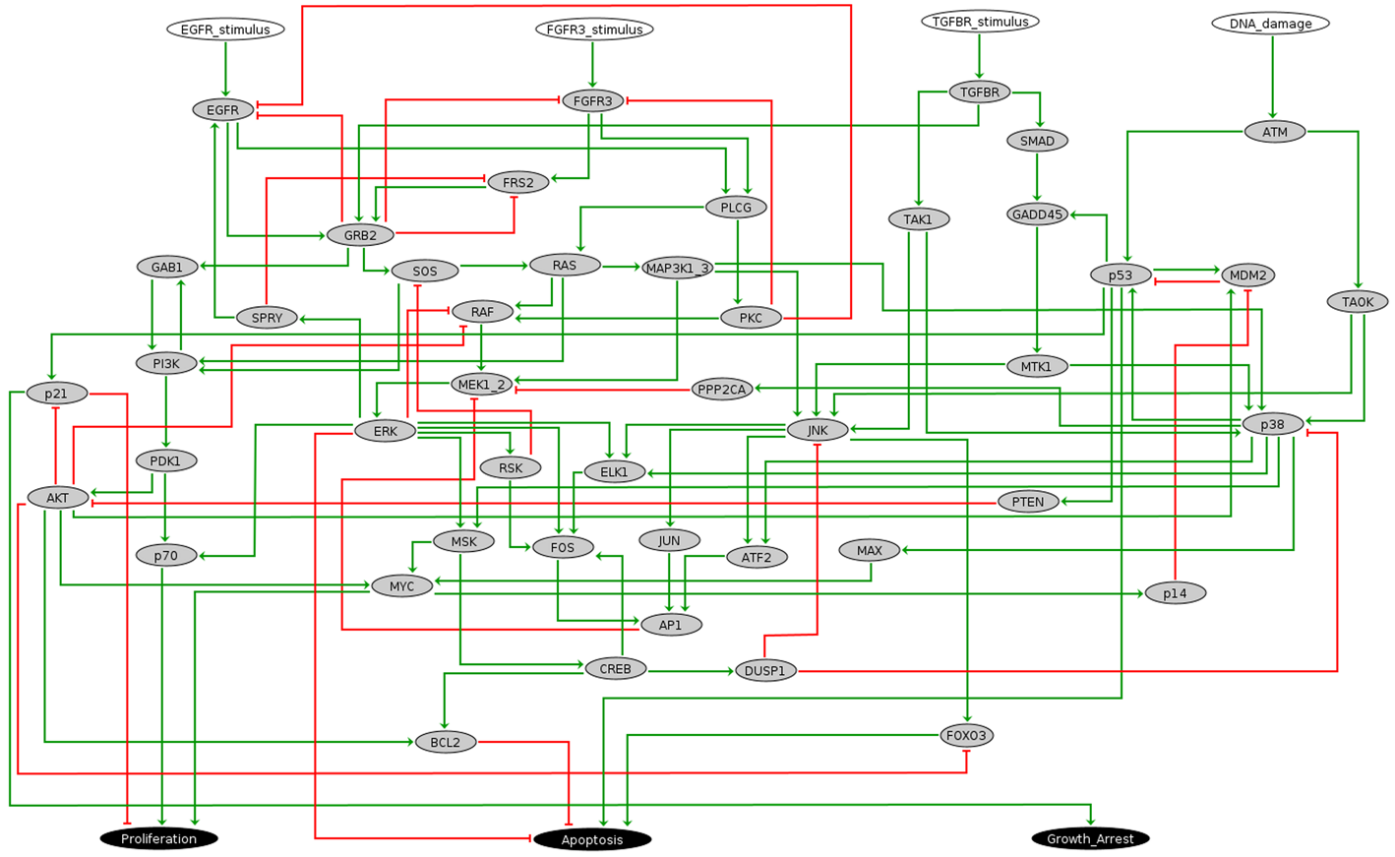
MAPK network presented in [2]. Figure adapted from Fig. 2 of [2], distributed under the terms of the Creative Commons Attribution License (http://creativecommons.org/licenses/by/3.0/). The model was loaded and the figure edited in GINsim [25]. Input and output nodes are colored in white and black respectively. Green edges denote activations and red edges denote inhibitions

We study the network introduced by Grieco et al. (2013) [2] to model the effect of the Mitogen-Activated Protein Kinase (MAPK) pathway on cell fate decisions in bladder cancer cells (see Fig. 5). The network consists of 53 Boolean variables, including the four inputs DNA-damage, EGFR-stimulus, FGFR3-stimulus and TGFBR-stimulus. The states of the three outputs of the network (Apoptosis, Growth-Arrest and Proliferation) indicate the enablement or disablement of the corresponding processes that represent the different cell fates or phenotypes considered in [2].

There are 18 attractors in the asynchronous dynamics, of which 12 are steady states and 6 are complex. The attractors are in one-to-one correspondence with the minimal trap spaces, that is, each attractor is contained in a minimal trap space and each minimal trap space only contains one attractor [16]. Therefore, we can use the selected trap spaces of Type 1 and Type 2 (see “Implementation” section) to search for control strategies via trap spaces.

In the first part, we target the subspace defined by the apoptotic phenotype and compare the control strategies identified via trap spaces to the ones by direct percolation, first for node control and then for edge control. In the second part, we consider the attractors of the asynchronous dynamics by targeting the minimal trap spaces. We compare the control strategies identified by direct percolation, via trap spaces and with the combination of the two methods (see Algorithm 1) for four steady states for node and edge control. In all the cases, we obtain new control strategies via trap spaces missed by direct percolation.

#### Target: apoptotic phenotype

We start by considering as target the apoptotic phenotype that is defined by the subspace obtained by fixing Apoptosis to 1, Growth-Arrest to 1 and Proliferation to 0 as in [2]. We refer to this subspace as the apoptotic target. We identify 103 non-percolating selected trap spaces. We set a limit size of three interventions.

In this setting, the combined method identifies 271 control strategies for node control up to size 3: three of size 1, 106 of size 2, 162 of size 3. The three control strategies of size 1 are $\{$(TGFBR-stimulus, 1)$\}$, $\{$(TGFBR, 1)$\}$ and $\{$(DNA-damage, 1)$\}$, the last one being obtained only via trap spaces. Under the control strategy $\{$(DNA-damage, 1)$\}$, the state space percolates to the trap space $\{$(ATM, 1), (DNA-damage, 1), (TAOK, 1)$\}$, which contains only attractors in the apoptotic target. This minimal control strategy is not identified by direct percolation. The number of control strategies identified by each method is shown in Table 1. As explained in the “Implementation” section, the list of control strategies obtained by the combination of the two methods might not be equal to the union of the control strategies obtained by each of the methods individually, since minimality is applied to each type of control strategy separately, and a control strategy that is minimal for one method might not be minimal under another method. In this case, there are eighteen control strategies of size 2 and thirteen of size 3 obtained by direct percolation that are supersets of the control strategy $\{$(DNA-damage, 1)$\}$. For example, $\{$(DNA-damage, 1), (SMAD, 1)$\}$ is identified as a control strategy by the direct percolation method since neither $\{$(DNA-damage, 1)$\}$ nor $\{$(SMAD, 1)$\}$ are control strategies by direct percolation. When considering the combination of both methods, $\{$(DNA-damage, 1)$\}$ is identified as a control strategy and consequently neither $\{$(DNA-damage, 1), (SMAD, 1)$\}$ nor any of the supersets of $\{$(DNA-damage, 1)$\}$ are considered. For this reason, there are fewer control strategies of size 2 and 3 for the combined method than by direct percolation (see Table 1). The method of direct percolation identifies many control strategies of size 2 and 3 that are missed by the method via trap spaces. These missed strategies fall into two categories. The first category includes the strategies that are not considered by the trap spaces method, since they include interventions targeting nodes not fixed in any selected trap space or fix them to the opposite value. For instance, the intervention set $\{$(DUSP1, 0), (PLCG,1)$\}$ is not taken into consideration since the node DUSP1 is not fixed to 0 in any of the selected trap spaces. The second category includes the sets of interventions that percolate directly to the target subspace but do not percolate to any of the selected trap spaces. For these reasons, it is useful to combine the method of direct percolation with the method via trap spaces to increase the total number of control strategies identified (see “Control strategies” for more details).

**Table 1:** Number and size of the control strategies identified by the different methods up to size 3 for the apoptotic target. Note that some minimal control strategies of size 2 and size 3 for direct percolation are not minimal for the combined method

Node control	$| \mathcal {N} | = 1$	$| \mathcal {N} | = 2$	$| \mathcal {N} | = 3$
By direct percolation	2	124	175
Via trap spaces	2	0	0
Combined	3	106	162

Edge control	$| \mathcal {E} | = 1$	$| \mathcal {E} | = 2$	$| \mathcal {E} | = 3$
By direct percolation	2	137	893
Via trap spaces	2	0	0
Combined	3	117	830

Node and edge control	$| \mathcal {C} | = 1$	$| \mathcal {C} | = 2$	$| \mathcal {C} | = 3$
By direct percolation	4	530	3569
Via trap spaces	4	0	0
Combined	6	454	3299

Using the combined method for edge control we obtain 950 control strategies up to size 3: three of size 1, 117 of size 2 and 830 of size 3 (see Table 1). The three edge control strategies of size 1 are equivalent to the node interventions identified as control strategies of size 1. This results from the three variables involved in the size 1 node control strategies having a unique incoming edge. For example (TGFBR-stimulus, TGFBR, 1) has exactly the same effect as (TGFBR, 1), since TGFBR is uniquely regulated by TGFBR-stimulus.

In other cases, edge control allows intervention strategies that would be too restrictive in node control. For example, the two edge interventions (MAP3 K1-3, p38, 1) and (MSK, CREB, 0), which fix the activation of MAP3 K1-3 in p38 and the inhibition of MSK in CREB, lead the controlled system to percolate to the apoptotic target. However, fixing MAP3 K1-3 to 1 and MSK to 0 does not, since the controlled system displays non-apoptotic steady states, which are not present in the original dynamics.

By allowing the combination of node and edge interventions, the combined method identifies over three thousand control strategies up to size 3, as shown in Table 1. Note that these include all the control strategies obtained for node and edge control. In particular, the six control strategies of size 1 correspond to the three control strategies of node control and the three of edge control.

We observe that there are many control strategies that mix node and edge interventions. Most of them include interventions already appearing in control strategies consisting exclusively of node interventions or of edge interventions. In some cases, we find mixed control strategies that are equivalent to a node control strategy or an edge control strategy where a node intervention is substituted by an equivalent edge intervention or vice versa. For example, the control strategy $\{$(CREB, DUSP1, 0), (TAOK, 1)$\}$ is equivalent to the control strategy $\{$(CREB, DUSP1, 0), (ATM, TAOK, 1)$\}$, since the node intervention (TAOK, 1) is equivalent to the edge intervention (ATM, TAOK, 1). There are also control strategies involving interventions that are not part of any node or edge control strategy. This is the case for $\{$(FGFR3, FRS2, 1), (GRB2, FRS2, 0), (p38, 1)$\}$, where neither (FGFR3, FRS2, 1) nor (GRB2, FRS2, 0) appear in any edge control strategy.

#### Target: minimal trap spaces

When computing control strategies for the minimal trap spaces, the input components need to be fixed in order to ensure that their value matches the one fixed in the target. Since each input combination identifies a separate trap space, there is at least one attractor per input combination. There are sixteen possible input combinations, fourteen of which identify subspaces that contain a unique attractor. These input combinations therefore give minimal node control strategies for the corresponding attractors. The subspaces induced by the two remaining input combinations ($\mathcal {S}_1 = \{$EGFR-stimulus = 0, FGFR3-stimulus = 0, TGFBR-stimulus = 0 and DNA-damage = 0$\}$, $\mathcal {S}_2 = \{$EGFR-stimulus = 0, FGFR3-stimulus = 0, TGFBR-stimulus = 0 and DNA-damage = 1$\}$) contain two steady states each and, therefore, further control interventions are needed. Table 2 shows the number and size of the control strategies up to size 7 (the number of inputs plus three) of these four steady states for node, edge and mixed control. Note that in all the cases there are control strategies identified via trap spaces not captured by direct percolation and there is no minimal control strategy identified by direct percolation missed via trap spaces. Moreover, no control strategy of size 5 is found for direct percolation for any of the steady states.

**Table 2:** Number and size of the control strategies identified by the different methods up to size 7 for the different steady states. Note that there is no control strategy up to size 4. $s_1$ and $s'_1$ denote the two steady states in $\mathcal {S}_1$ and $s_2$ and $s'_2$ the two steady states in $\mathcal {S}_2$

	$s_1$			$s'_1$			$s_2$			$s'_2$		
Node control												
$| \mathcal {N}|$	5	6	7	5	6	7	5	6	7	5	6	7
By direct percolation	0	0	60	0	0	32	0	14	2	0	14	2
Via trap spaces	2	0	0	0	8	12	0	22	14	2	0	0
Combined	2	0	0	0	8	12	0	22	14	2	0	0
Edge control												
$| \mathcal {E}|$	5	6	7	5	6	7	5	6	7	5	6	7
By direct percolation	0	0	150	0	0	84	0	22	58	0	33	50
Via trap spaces	3	1	0	0	14	20	0	36	64	3	1	0
Combined	3	1	0	0	14	20	0	36	64	3	1	0
Node and edge control												
$| \mathcal {C}|$	5	6	7	5	6	7	5	6	7	5	6	7
By direct percolation	0	0	12720	0	0	7040	0	1168	2608	0	1440	2048
Via trap spaces	80	16	0	0	704	2112	0	1872	4192	80	16	0
Combined	80	16	0	0	704	2112	0	1872	4192	880	16	0

As in the case of the apoptotic target, there are edge control strategies allowing interventions that would not be possible using only node control. For example fixing the component GRB2 either to 0 or to 1, together with the corresponding input interventions, does not lead to a system with $s_1$ as the unique attractor. However, fixing GRB2 in the edge intervention (GRB2, GAB1, 1) in addition to the input interventions leads to a controlled dynamics that has $s_1$ as a unique attractor.

When considering mixed interventions, in contrast to the apoptotic target case, all the interventions appearing in minimal control strategies also occur in some strategy composed exclusively of node or exclusively of edge interventions. As can be seen from the numbers in Table 2, we still gain many mixed control strategies and thus more flexibility for choosing interventions that are both realizable in the lab and as non-invasive as possible for the system.

#### Running times

The running times of the control strategy computation for each method, target and type of control are shown in Table 3. These refer to the total times needed for Algorithm 1 to terminate for each method, including the computation of the selected trap spaces when needed. We can observe how the number of candidate interventions affects the time required for each method. Node control is the fastest, around a few centiseconds, whereas edge control requires a few seconds. The running times of the different methods vary from a few seconds to a few minutes when combining the two types of interventions. Although the apoptotic phenotype is the target with the highest number of selected trap spaces, we do not observe a significant increase of the running time with respect to the steady states. This could perhaps result from the additional constraint on candidate interventions that is imposed when working with selected trap spaces, requiring the interventions to be selected among the variables fixed in the trap space.

All the results presented in this section were obtained with a regular desktop 8-processor computer, Intel^®^Core^TM^ i7-2600 CPU at 3.40GHz, 16GB memory.

**Table 3:** Running times (in seconds) for the control strategy computation targeting the apoptotic phenotype and the four steady states in the MAPK network. The size of the target is defined as the number of fixed components in the target subspace

Target	Size of	Number of selected	Method	Time (s)		
	The target	Trap spaces		Node	Edge	Both
Apoptotic phenotype	3	103	By direct percolation	0.18	7.05	280.18
			Via trap spaces	0.47	0.82	0.74
			Combined	7.15	117.36	461.37
Steady state $s_1$	53	3	By direct percolation	0.04	8.41	125.65
			Via trap spaces	0.07	7.39	289.18
			Combined	0.08	17.07	149.47
Steady state $s'_1$	53	3	By direct percolation	0.04	1.61	154.79
			Via trap spaces	0.09	1.40	104.80
			Combined	0.15	4.69	211.49
Steady state $s_2$	53	2	By direct percolation	0.03	0.42	10.71
			Via trap spaces	0.10	1.03	33.33
			Combined	0.10	3.26	155.31
Steady state $s'_2$	53	2	Direct percolation	0.03	1.25	12.29
			Via trap spaces	0.10	1.87	6.56
			Combined	0.11	3.60	12.31

### Comparison

The approach via trap spaces (TS), the direct percolation approach (DP) and the combined method (CM) are compared to two different control strategy identification methods for attractor control: the stable-motifs approach (SM) and the basin-based approach (BA). An overview of the features of every method is shown in Table 4. The stable-motifs approach is based on the identification of the network stable motifs as described in [5]. The basin-based approach computes the basins of attraction of the network attractors in order to identify control strategies of minimum size as explained in [14]. The stable motifs of the network are related to the trap spaces of the Boolean function, since they capture components locking each other to certain values, so it is reasonable that similar control strategies are obtained by TS and SM in most of the cases. The computation of the basins of attraction, on the other hand, provides full information about the network dynamics, therefore BA should be able to identify all the control strategies of minimum size.

We compare the control strategies that use permanent interventions, since these are the type of perturbations considered in Definition 1 and, consequently, used by DP, TS and CM. The control strategies obtained by TS and SM can be applied using either permanent or temporary perturbations, since they lead the dynamics to attractors of the original network. CABEAN software can identify different control strategies depending on the type of perturbations (permanent, temporary or instant) that is selected. For this comparison, we run CABEAN selecting the option of permanent perturbations. Both BA and SM only consider node control in the asynchronous update. Thus, the comparison is only made for permanent node control in this dynamics. Although some methods for target control can deal with edge interventions [9], they only require that the steady states of the controlled system belong to the target subspace, and impose no restrictions on the complex attractors that might also exist. Since our approach identifies control strategies that require that all attractors (steady states or complex) belong to the target, a direct comparison to these methods is not possible.

**Table 4:** Overview of the versatility of the different control methods in terms of the types of targets, interventions and update schemes

Method		Tool	Control target				Update			Interv.	
			Steadystate	Trap spaceattractor	Complexattractor	Subspace	Async.	Sync.	Gen. async.	Node	Edge
Basins of attr.	BA	CABEAN [12]	$\checkmark$	$\checkmark$	$\checkmark$	–	$\checkmark$	–	–	$\checkmark$	–
Stable motifs	SM	StableMotifs [5]	$\checkmark$	$\checkmark$	–	–	$\checkmark$	–	–	$\checkmark$	–
Direct perc.	DP	pyboolnet	$\checkmark$	$\checkmark$	–	$\checkmark$	$\checkmark$	$\checkmark$	$\checkmark$	$\checkmark$	$\checkmark$
Trap spaces	TS	pyboolnet	$\checkmark$	$\checkmark$	–	$\checkmark$	$\checkmark$	$\checkmark$	$\checkmark$	$\checkmark$	$\checkmark$
Combined	CM	pyboolnet	$\checkmark$	$\checkmark$	–	$\checkmark$	$\checkmark$	$\checkmark$	$\checkmark$	$\checkmark$	$\checkmark$

We consider three biological networks of different sizes with different type and number of attractors. The first one is the MAPK network, already introduced in the previous section. The second is the cell-fate network, introduced by Calzone et al. (2010) [24] to model cell fate decision processes. It consists of 28 Boolean variables and has 27 attractors in the asynchronous dynamics, all of them steady states. The third network is the T-LGL network, originally introduced by Zhang et al. (2008) [3] to model the T cell large granular lymphocyte (T-LGL) survival signaling network, which we use in the version presented in [5]. This network consists of 60 Boolean variables and has 3 complex attractors in the asynchronous dynamics. Table 5 shows an overview of the three networks and their features. As mentioned in the “Implementation” section, our method takes as input a constant-free Boolean function. Therefore, since the input variables of the T-LGL network are fixed, the constant values are percolated and removed from the network. In this case, for the sake of the comparison, the reduced network is used as input for all the methods. The complete Boolean rules of each network can be found in the repository of PyBoolNet [22].

**Table 5:** Main features of the biological networks used in the comparison. The input variables are fixed in the T-LGL network and free in the cell-fate and MAPK networks

Network		Size	Inputs	Outputs	Attractors	
					Steady	Cyclic
Cell-fate	[24]	28	3	3	27	0
MAPK	[2]	53	4	3	12	6
T-LGL	[3]	60	6	3	0	3

For each network, we choose one of its attractors as the control target. To enrich the comparison, we select attractors for which the control strategies obtained by each method differ. The control target for the T-LGL network is the apoptotic attractor, that is, the attractor with Apoptosis = 1 and Proliferation = 0. This is the only attractor where the control strategies obtained for SM, BA and TS differ. The three methods identify the same control strategies for the other two attractors. The target attractor for the MAPK network is $s_2$ (see “Target: minimal trap spaces”). Both SM and TS obtain the same control strategies for the remaining attractors of the MAPK network. The results for BA could not be obtained since the software was not able to process this network. The control target for the cell-fate network is the apoptotic steady state (Apoptosis = 1) that has the three inputs (FADD, FASL and TNF) set to 1. It is one of the ten attractors for which TS identifies more control strategies than SM. In the remaining seventeen attractors the two methods obtain the same control strategies. TS identifies more control strategies than BA in eighteen attractors, including the selected steady state.

Table 6 shows the number and size of the control strategies identified by each method for the selected attractors. In the MAPK and cell-fate networks, TS is able to identify all the control strategies identified by SM and BA and, in some cases, new control strategies missed by the other methods. In the third network, TS is able to identify three of the four minimal control strategies identified by the other methods $\{$(SPHK1, 0)$\}$, $\{$(PDGFR, 0)$\}$ and $\{$(S1P, 0)$\}$. It misses the fourth one $\{$(Ceramide, 1)$\}$ identifying, instead, five non-minimal control strategies combining (Ceramide, 1) with (PLCG1, 1), (GRB2, 1), (IL2RBT, 1), (IL2RB, 1) and (RAS, 1) respectively. Note that no control strategy for this attractor up to size 2 is identified by direct percolation.

**Table 6:** Number and size of the control strategies for the corresponding attractor identified by the different methods up to sizes 6, 5 and 2 (number of free inputs plus two) for the networks MAPK, cell-fate and T-LGL respectively

	MAPK network		Cell-fate network		T-LGL network	
	$| \mathcal {N}| \le 5$	$| \mathcal {N}| = 6$	$| \mathcal {N}| \le 4$	$| \mathcal {N}| = 5$	$| \mathcal {N}| = 1$	$| \mathcal {N}| = 2$
SM	0	16	0	16	3	0
BA	–	–	0	15	4	0
DP	0	14	0	22	0	0
TS	0	22	0	22	3	5
CM	0	22	0	22	3	5

The running times for each method are shown in Table 7. They include the time for reading the network, computing the attractors or minimal trap spaces and identifying the control strategies. As in the previous section, all the results were obtained with a regular desktop 8-processor computer, Intel^®^Core^TM^ i7-2600 CPU at 3.40GHz, 16GB memory. Note that while BA and TS allow the selection of a specific target (attractor in case of BA, subspace in case of TS), SM computes the control strategies for all the attractors in one run. For this reason, in order to have a fair comparison, we show the running times for the computation of the control strategies for all the attractors of each network. Moreover, since we could not fix a limit size for the control strategies in SM and BA, we also did not set a limit size for DP, TS and CM. It is worth noting that the running time of BA in the cell-fate network is very dependent on the attractor chosen. In particular, for three of the attractors BA takes over one hour each, whereas for the other attractors the computation time is of the order of seconds. In all the cases we see that the running times for DP, TS and CM are significantly smaller than for BA and SM.

**Table 7:** Running times for the control strategy computation of each method for the three networks. The time shown includes the computation of the system attractors or minimal trap spaces and of the control strategies targeting every attractor or minimal trap space

Method		Tool	Running times		
			Cell-fate	MAPK	T-LGL
Stable motifs	SM	StableMotifs [5]	4 h54’58”	3’14”	5’56”
Basins of attraction	BA	CABEAN [12]	3 h30’52”	-	2’10”
Direct percolation	DP	pyboolnet	1.4”	1.9”	1.2”
Via trap spaces	TS	pyboolnet	2.4”	2.2”	1.4”
Combined: DP+TS	CM	pyboolnet	2.4”	2.2”	1.4”

## Discussion

In this work we deal with node and edge interventions both separately and combined. When mixing the two types of interventions, it is necessary to define a priority order to avoid the inconsistency problems that might arise. Here we consider that node control takes priority over edge control and we forbid contradictory interventions targeting the same component. Further works could include different prioritisation orders and study how these might affect the controllability of a system. Another aspect that needs careful consideration is the definition of optimality for control strategies that can include both node and edge interventions. Here we considered minimality with respect to inclusion exclusively. Specific evaluations of the costs required to implement different control interventions could lead to the formulation of optimization functions more fitting to the specific model.

The formulation of these control problems as Boolean constraint problems in Answer Set Programming (ASP), extending the works from [15] and [18], aims to address the challenge of their associated combinatorial explosion. While our implementation can handle state-of-the-art biological models, further experiments are required to fully evaluate the scalability of our extended implementation. Although in biological interaction networks the number of regulators for each component is often small in comparison to the overall number of species, which significantly helps in limiting the computational load, topological properties of the network can have a substantial impact. In particular, our approach requires the identification of some trap spaces (selected trap spaces), which are used as inputs for the ASP program. The number of these selected trap spaces can be relatively high, for instance in the case of networks with many steady states, and significantly impact the running times. On the other hand, constraint programs for different selected trap spaces could be solved in parallel, with a post-processing step to ensure minimality of the results. An alternative approach could aim at identifying the relevant selected trap spaces by extending the constraint problem, rather than as a preliminary step, avoiding the costly explicit enumeration. In addition, all experiments were run using *clingo* default settings [23]; performance analysis and tuning could yield improved running times.

The use of selected trap spaces could be easily extended to other types of control. A control strategy that drives the dynamics to a selected trap space can be seen as a *transient* control strategy, meaning that the intervention could be applied for a certain period of time, until the trap space is reached, and then be released. We think that trap spaces could be further exploited for the identification of more sophisticated control approaches, like sequential interventions. Given the flexibility and efficacy shown by constraint-based approaches, the extension to these problems, in particular in ASP, should be explored.

## Conclusions

The method presented in this work provides a new tool for control strategy identification, based on value percolation, that uses trap spaces to identify potentially smaller control strategies that could be missed by usual percolation-based methods. This approach implements the standard node interventions acting on specific components, as well as edge interventions acting on interactions between them. Considering edge interventions widens the range of possible control strategies, for example when restrictions on the components that can be subject to intervention prevents the applicability of node control for a desired target. It can also broaden the possibilities for potential applications, for instance by allowing to act on the specific interaction between two proteins, while preserving their role in other potentially critical cell processes. The examples of edge control strategies shown in the MAPK case study illustrate the diverse and new possibilities offered by edge control.

## Methods

### Problem encoding

In this section we explain in more detail the ASP encoding used for control strategy identification. We recall the work done in [15] and [18] to identify the control strategies presented in [8] and extend it to deal with edge control. This implementation uses *clingo*, which consists of the grounder *gringo* and the solver *clasp*, included in Potassco, the Potsdam Answer Set Solving Collection [23]. Therefore, our explanations about ASP syntax and semantics are based on the language used by *clingo*. For a more extensive and detailed explanation of Answer Set Programming and its semantics we refer the reader to [21].

The ASP encoding consists of two different parts: the encoding of the control problem (program instance) and the encoding of the computation process (main program). The encoding of the control problem includes the Boolean function, assumed to be given in disjunctive normal form (DNF), the target subspaces and selected trap spaces, the limit size of the control strategies and the restrictions on the nodes and edges that can be used for control.

**Fig. 6:**
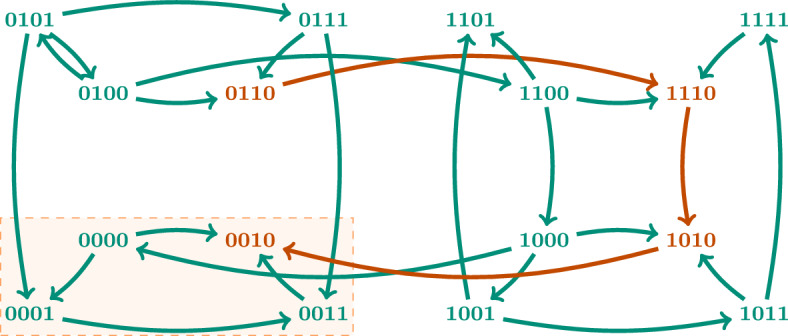
The asynchronous dynamics of the Boolean function $f(x) = (x_2 {\bar{x}}_4 \vee x_1 x_4 \vee x_1 x_2$, ${\bar{x}}_1 x_2 {\bar{x}}_4 \vee x_1 x_4$, $x_3 \vee {\bar{x}}_4 \vee {\bar{x}}_2 \vee {\bar{x}}_1$, ${\bar{x}}_4 {\bar{x}}_3 \vee {\bar{x}}_2 {\bar{x}}_3 \vee x_1 {\bar{x}}_3)$ has two attractors $\mathcal {A}_1 = \{0010\}$ and $\mathcal {A}_2 = \{1101\}$. The selected trap spaces of *f* containing only attractors in the target subspace $P = 00{*}{*}$ (orange) are $T_1 = {*}{*}10$ (red) and $T_2 = 0010$ (the first steady state). $\mathbb {B}^n$ percolates to the selected trap space $T_1$ under the controlled functions $f^{\mathcal {N}_1}(x) = (x_1 x_2 \vee x_2$, ${\bar{x}}_1 x_2$, 1, 0), with $N_1 = \{(4,0)\}$, and $f^{\mathcal {N}_2}(x) = (x_2 {\bar{x}}_4 \vee x_1 x_2 \vee x_1 x_4$, $x_1 x_4 \vee {\bar{x}}_1 x_2 {\bar{x}}_4$, 1, 0), with $N_2 = \{(3,1)\}$. Example adapted from Fig. 1 of [18], distributed under the terms of the Creative Commons Attribution license (http://creativecommons.org/licenses/by/4.0/)

The Boolean function is encoded as described in [15]. We allow the possibility of excluding certain interventions from the control candidates, for instance in the case that an intervention is not feasible for application. The node and edge interventions that we want to exclude from the control are declared in the literals avoid$\_$node or avoid$\_$edge respectively (line 1). The Boolean network from the example in Fig. 6 is encoded as follows. The literal formula (line 3) links every variable with its DNF, described by the literals dnf and clause (lines 4–11). The regulatory function of the first component $f_1(x) = x_2 {\bar{x}}_4 \vee x_1 x_2 \vee x_1 x_4$ is declared in the literal formula(x1, 0) (line 3) and linked to its three clauses dnf(0, 0), dnf(0, 1) and dnf(0, 2) (line 4). The first clause $x_2 {\bar{x}}_4$ is encoded in the literals clause(0, x2, 1) and clause(0, x4, −1) (line 6). Note that we use−1 and 1 in the third variable of the literal clause to denote whether a variable is negated or not, respectively. To ease the encoding, we also use the value−1 to represent the Boolean value 0 in the rest of the program. Note that all the rules of the program instance are *facts* that describe the control problem.
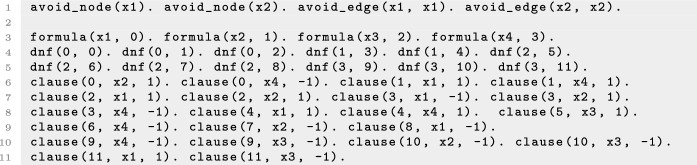


The target subspace and the target trap spaces are encoded in the literal subspace (line 12). We use two types of identifier: positive and negative. The positive identifier marks the subspace as a selected trap space and the negative identifier marks it as the direct target. The fixed variables of each subspace are encoded in the variable goal (lines 13–14) as in [15] and [18]. The selected trap space ${*}{*}10$ from Fig. 6 is encoded by the variables subspace(1), goal(1, x3, 1) and goal(1, x4,−1) (lines 12–13). A limit size on the number of interventions is set in line 16.
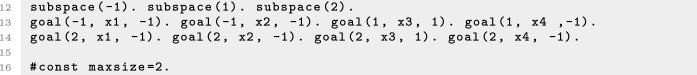


The main ASP program for control strategy identification can be divided in four parts: candidate instantiation, new controlled function instantiation (only necessary for edge interventions), percolation step and satisfaction requirements. The first two parts differ in node and edge control whereas the last two are the same. In the following, we describe each step in detail.

The candidate instantiation for node control is adapted from [15] as described in [18]. Lines 1–5 instantiate the literals node(V,S) for direct percolation as described in [15]. Note that, in order to reduce the number of candidates, a candidate node intervention node(V, S) for direct percolation is instantiated only when there is a positive (respectively negative) path from the node V to one of the nodes fixed in the target subspace to the value S (respectively-S). See [15] and [6] for a full explanation of this reduction. The conditions satisfied(Z), Z < 0 are added to the rule in line 5 to guarantee that these node interventions are only instantiated when the state space percolates to the target, marked with a negative identifier. Line 6 instantiates the candidate interventions for control via trap spaces. When the state space percolates to a selected trap space, it is necessary that the candidate interventions are chosen among the variables fixed in the trap space. This is ensured in line 6. In this case, the conditions satisfied(Z), Z > 0 are added in line 6 to guarantee that these node interventions are only instantiated when the state space percolates to the selected trap space identified by Z. In the example of Fig. 6, the instantiations of the fixed variables of the selected trap space $T = {*}{*}10$ are goal(1, x3, 1) and goal(1, x4, −1). Consequently, the node interventions considered for control via this trap space are node(x3, 1) and node(x4, −1). Line 7 excludes contradictory node interventions, that is, two node interventions fixing the same node to opposite values. In line 8, the variable node(V) is declared to keep track of the controlled nodes.
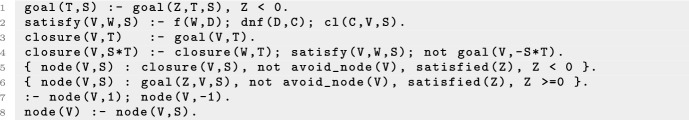


The candidate instantiation for edge control is shown below. First, the candidate edge interventions are generated (lines 9–10). Note that the existence of a clause involving *i* in the DNF of $f_j$ is required in order to instantiate the candidate edge intervention (*i*, *j*, *c*) and that forbidden edges are excluded. It is also ensured that, when the state space percolates to a selected trap space, the edge interventions target the variables fixed in the trap space (line 10). Contradictory edge interventions are also excluded (line 12). We keep track of the controlled edges with the variable edge(Vi,Vj) (line 13).
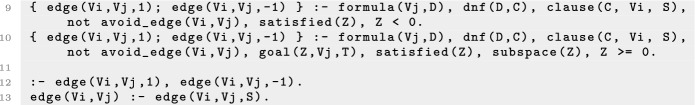


When considering node and edge interventions together, the following restrictions are also added, to ensure that the set of interventions is consistent. They prevent that a node and an edge intervention fix or target the same variable (lines 15 and 16 respectively).
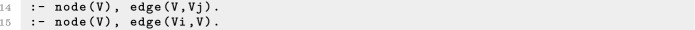


In order to capture the effect of the edge interventions in the Boolean function, a new set of literals new$\_$ clause, new$\_$ dnf, new$\_$ formula is instantiated to represent the DNF of the resulting regulatory functions (lines 16–20). These new literals are directly instantiated for every term, clause and DNF respectively that are not affected by edge interventions. When a clause is affected by an edge intervention, there are two possible situations. The edge intervention fixes the term to 1, in which case the term just disappears from the clause, or it fixes the term to 0, in which case the whole clause is evaluated to 0. In the first situation, the term is just not included in the new clause, that is, the corresponding new$\_$ clause literal is not instantiated. In the second situation, the clause needs to be removed from the DNF so the literal remove$\_$ dnf is instantiated to indicate that this clause should be removed (line 17). Line 18 links all the clauses that do not need to be removed to the new DNF. Note that if all the terms of a clause get fixed to 1, the whole DNF is set to 1. In this case, the literal remove$\_$ formula is instantiated (line 19) so that the new$\_$ formula literal is not generated, since the regulatory function is constant. To indicate that the component V is set to 1, the literal fixed$\_$ node(V,1) is also instantiated (line 22). If all the clauses of a DNF are evaluated to 0 and consequently removed from the disjunction, the regulatory function becomes the constant 0. Then, the literal new$\_$ formula is not instantiated and the literal fixed$\_$ node(V,−1) is generated to indicate that the component V is set to 0 (line 23). When none of the non-empty DNF clauses are evaluated to one, the literal new$\_$ formula is instantiated to link them to the corresponding regulatory function (line 19).
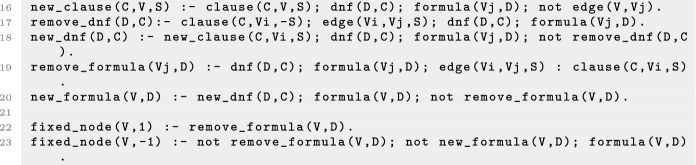


Let us consider the case of the edge intervention edge(x4,x3,1), that fixes $x_4 = 1$ in $f_3$. Since $f_3(x) = {\bar{x}}_1 \vee {\bar{x}}_2 \vee {\bar{x}}_4 \vee x_3$, fixing $x_4 = 1$ leads to the clause ${\bar{x}}_4$ being evaluated to 0 and the regulatory function becomes $f^{(4,3,1)}_3(x) = {\bar{x}}_1 \vee {\bar{x}}_2 \vee x_3$ (the same without the clause ${\bar{x}}_4$). The ASP program will instantiate the literals new$\_$ clause(5,x3,1), new$\_$ clause(7,x2,−1), new$\_$ clause(8,x1,−1) since they are not affected by the edge intervention. It will also instantiate the literals new$\_$ dnf(2,5), new$\_$ dnf(2,7), new$\_$ dnf(2,8) that connect the clauses to the new DNF of $f_3$ and remove$\_$ dnf(2,6), since the edge intervention edge(x4,x3,1) and the term in the clause clause(6,x4,−1) have opposite signs. Although new$\_$ dnf(2,6) will not be instantiated, new$\_$ formula(x3,2) will, since its DNF will still have the non-trivial clauses new$\_$ dnf(2,5), new$\_$ dnf(2,7), new$\_$ dnf(2,8).

If instead the edge intervention was edge(x4,x3,−1), fixing $x_4 = 0$ in $f_3$ would set the regulatory function to 1 ($f^{(4,3,0)}_3(x) = 1$). In this case, remove$\_$ formula(x3,2) would also be instantiated, since there would be a clause in the DNF that gets evaluated to 1, and new$\_$ formula(x3,2) will not, since the DNF would become 1.

The regulatory functions that become constants either through node or edge control are captured in the literals intervention(V,S) (lines 24 and 25 respectively) and tracked by the literal intervention(V) (line 26).
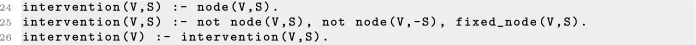


The percolation effect is then encoded in the same way as described in [15] using the literal intervention(V,S) (lines 27–31). See [15] for a full explanation.
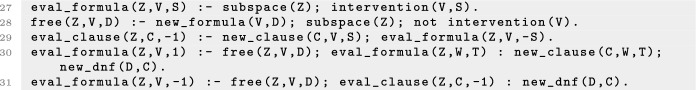


Finally, it is ensured that a candidate subspace is a control strategy as described in [18], by requiring that at least one subspace constraint is satisfied (lines 32–34). A subspace constraint is satisfied when all the variables that are fixed in the subspace have their regulatory functions fixed after the percolation step. This is checked in line 33 by stating that a subspace Z is satisfied if for all goal(Z,T,S) the regulatory corresponding to component T has been fixed to S, that is, if the literal eval$\_$ formula(Z,T,S) is true. A limitation on the total number of interventions is also added (line 35).
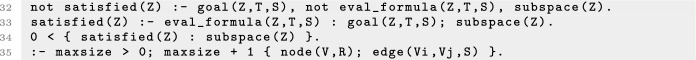

**Algorithm 1:**
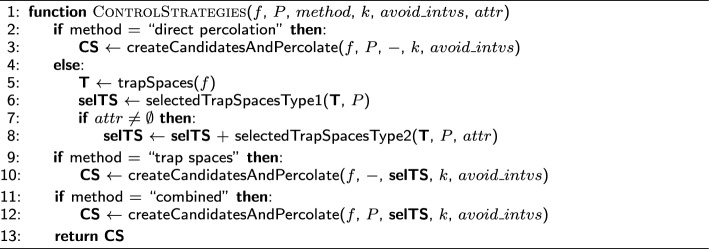
Control strategies for a target subspace

## Data Availability

The Boolean networks analysed in this work are available in the PyBoolNet repository, https://github.com/hklarner/pyboolnet/blob/master/pyboolnet/repository/. The source code of the implementation is available at https://github.com/Lauracf/trap-space-control.

## Abbreviations

ASP: Answer set programming
MAPK: Mitogen-activated protein kinase
T-LGL: T cell large granular lymphocyte
DP: Direct percolation approach
TS: Via trap spaces approach
CM: Combined approach
SM: Stable-motifs approach
BA: Basins of attraction approach
DNF: Disjunctive normal form

## About this supplement

This article has been published as part of BMC Bioinformatics Volume 24 Supplement 1, 2023: Special Issue of the 19th International Conference on Computational Methods in Systems Biology. The full contents of the supplement are available online at https://bmcbioinformatics.biomedcentral.com/articles/supplements/volume-24-supplement-1.

## References

[1] Flobak Å, Baudot A, Remy E, Thommesen L, Thieffry D, Kuiper M, Lægreid A. Discovery of drug synergies in gastric cancer cells predicted by logical modeling. PLoS Comput Biol. 2015;11(8):1–20. 10.1371/journal.pcbi.1004426. 10.1371/journal.pcbi.1004426 PMC4567168 26317215

[2] Grieco L, Calzone L, Bernard-Pierrot I, Radvanyi F, Kahn-Perlès B, Thieffry D. Integrative modelling of the influence of MAPK network on cancer cell fate decision. PLoS Comput Biol. 2013;9(10):1–15. 10.1371/journal.pcbi.1003286. 10.1371/journal.pcbi.1003286 PMC3821540 24250280

[3] Zhang R, Shah MV, Yang J, Nyland SB, Liu X, Yun JK, Albert R, Loughran TP. Network model of survival signaling in large granular lymphocyte leukemia. Proc Natl Acad Sci. 2008;105(42):16308–13. 10.1073/pnas.0806447105. 18852469 10.1073/pnas.0806447105 PMC2571012

[4] Mandon H, Su C, Haar S, Pang J, Paulevé L. Sequential reprogramming of Boolean networks made practical. In: Bortolussi L, Sanguinetti G, editors. Computational Methods in Systems Biology. Cham: Springer; 2019. p. 3–19. 10.1109/TCBB.2019.2914383 31056515

[5] Zañudo JGT, Albert R. Cell fate reprogramming by control of intracellular network dynamics. PLoS Comput Biol. 2015;11(4):1–24. 10.1371/journal.pcbi.1004193. 10.1371/journal.pcbi.1004193 PMC4388852 25849586

[6] Samaga R, Kamp AV, Klamt S. Computing combinatorial intervention strategies and failure modes in signaling networks. J Comput Biol. 2010;17(1):39–53. 10.1089/cmb.2009.0121. 20078396 10.1089/cmb.2009.0121

[7] Yang G, Gómez Zañudo, Tejeda J, Albert R. Target control in logical models using the domain of influence of nodes. Front Physiol. 2018;9:454. 10.3389/fphys.2018.00454. 29867523 10.3389/fphys.2018.00454 PMC5951947

[8] Cifuentes Fontanals L, Tonello E, Siebert H. Control strategy identification via trap spaces in Boolean networks. In: Abate, A., Petrov, T., Wolf, V. (eds.) Computational Methods in Systems Biology, pp. 159–175. Springer, Cham (2020). 10.1007/978-3-030-60327-4_9

[9] Biane C, Delaplace F. Causal reasoning on Boolean control networks based on abduction: theory and application to cancer drug discovery. IEEE/ACM Trans Comput Biol Bioinf. 2019;16(5):1574–85. 10.1109/TCBB.2018.2889102. 10.1109/TCBB.2018.2889102 30582550

[10] Murrugarra D, Veliz-Cuba A, Aguilar B, Laubenbacher R. Identification of control targets in Boolean molecular network models via computational algebra. BMC Syst Biol. 2016;10(1):94. 10.1186/s12918-016-0332-x. 27662842 10.1186/s12918-016-0332-x PMC5035508

[11] Sordo Vieira L, Laubenbacher RC, Murrugarra D. Control of intracellular molecular networks using algebraic methods. Bull Math Biol. 2020. 10.1007/s11538-019-00679-w. 10.1007/s11538-019-00679-w PMC8177064 31919596

[12] Su C, Pang J. CABEAN: a software for the control of asynchronous Boolean networks. Bioinformatics. 2020;37(6):879–81. 10.1093/bioinformatics/btaa752. 10.1093/bioinformatics/btaa752 32845335

[13] Cifuentes-Fontanals L, Tonello E, Siebert H. Control in Boolean networks with model checking. Front Appl Math Stat. 2022. 10.3389/fams.2022.838546.

[14] Su C, Pang J. Target control of asynchronous Boolean networks. IEEE/ACM Trans Comput Biol Bioinf. 2021. 10.1109/TCBB.2021.3133608. 10.1109/TCBB.2021.3133608 34882560

[15] Kaminski R, Schaub T, Siegel A, Videla S. Minimal intervention strategies in logical signaling networks with ASP. Theory Pract Logic Program. 2013;13(4–5):675–90. 10.1017/S1471068413000422.

[16] Klarner H, Siebert H. Approximating attractors of Boolean networks by iterative CTL model checking. Front Bioeng Biotechnol. 2015;3:130. 10.3389/fbioe.2015.00130. 26442247 10.3389/fbioe.2015.00130 PMC4562258

[17] Klarner H, Bockmayr A, Siebert H. Computing maximal and minimal trap spaces of Boolean networks. Nat Comput. 2015;14:535–44. 10.1007/s11047-015-9520-7.

[18] Cifuentes Fontanals L, Tonello E, Siebert H. Computing trap space-based control strategies for Boolean networks using answer set programming. AIP Conf Proc. 2022;2611(1):110002. 10.1063/5.0122073.

[19] Cifuentes Fontanals L. Methods for control strategy identification in Boolean networks. PhD thesis, Freie Universität Berlin, Mathematics and Computer Science Department 2022

[20] Zhong Q, Simonis N, Li Q-R, Charloteaux B, Heuze F, Klitgord N, Tam S, Yu H, Venkatesan K, Mou D, Swearingen V, Yildirim MA, Yan H, Dricot A, Szeto D, Lin C, Hao T, Fan C, Milstein S, Dupuy D, Brasseur R, Hill DE, Cusick ME, Vidal M. Edgetic perturbation models of human inherited disorders. Mol Syst Biol. 2009;5(1):321. 10.1038/msb.2009.80. 19888216 10.1038/msb.2009.80 PMC2795474

[21] Gebser M, Kaminski R, Kaufmann B, Schaub T. Answer set solving in practice. Synthesis Lectures on Artificial Intelligence and Machine Learning. Morgan & Claypool Publishers; 2013.

[22] Klarner H, Streck A, Siebert H. PyBoolNet: a Python package for the generation, analysis and visualization of Boolean networks. Bioinformatics. 2016;33(5):770–2. 10.1093/bioinformatics/btw682. 10.1093/bioinformatics/btw682 27797783

[23] Gebser M, Kaufmann B, Kaminski R, Ostrowski M, Schaub T, Schneider M. Potassco: the Potsdam Answer Set Solving Collection. AI Commun. 2011;24(2):107–24.

[24] Calzone L, Tournier L, Fourquet S, Thieffry D, Zhivotovsky B, Barillot E, Zinovyev A. Mathematical modelling of cell-fate decision in response to death receptor engagement. PLoS Comput Biol. 2010;6(3):1–15. 10.1371/journal.pcbi.1000702. 10.1371/journal.pcbi.1000702 PMC2832675 20221256

[25] Chaouiya C, Naldi A, Thieffry D. Logical Modelling of Gene Regulatory Networks with GINsim. 2012;804:463–79. 10.1007/978-1-61779-361-5_23. 10.1007/978-1-61779-361-5_23 22144167

